# Impact of brain atrophy on 90-day functional outcome after moderate-volume basal ganglia hemorrhage

**DOI:** 10.1038/s41598-018-22916-3

**Published:** 2018-03-19

**Authors:** Sae Min Kwon, Kyu-Sun Choi, Hyeong-Joong Yi, Yong Ko, Young-Soo Kim, Koang-Hum Bak, Hyoung-Joon Chun, Young-Jun Lee, Ji Young Lee

**Affiliations:** 1 Department of Neurosurgery, College of Medicine, Hanyang University, Seoul, Korea; 2 Department of Radiology, College of Medicine, Hanyang University, Seoul, Korea

## Abstract

This study aimed to evaluate the effect of brain atrophy on the functional outcome of patients with moderate-volume basal ganglia hemorrhage. Of 1003 patients with spontaneous intracerebral hemorrhage, 124 with moderate-volume basal ganglia hemorrhage (hematoma volume of 20–50 mL) were enrolled. The intercaudate distance (ICD) and sylvian fissure ratio (SFR) were used as linear brain atrophy parameters. The patients were divided into groups with favorable and unfavorable outcomes, according to the Glasgow Outcome Scale score, 90 days after symptom onset. Demographic and radiographic features, including the ICD and SFR, were compared between the two groups. Among the 124 patients, 74 (59.7%) exhibited a favorable outcome. The ICD and SFR values were significantly greater for the favorable group than for the unfavorable group. Multivariate analysis indicated that young age, high Glasgow Coma Scale score at admission, small hematoma volume, and increased ICD (odds ratio [OR], 1.207; 95% confidence interval [CI], 1.004–1.451) and SFR (OR, 1.046; 95% CI, 1.007–1.086, per 0.001) values had a beneficial effect on functional outcome. In conclusion, brain atrophy exhibits protective effects in patients with moderate-volume basal ganglia hemorrhage, and is an important factor for predicting functional outcome.

## Introduction

Spontaneous intracerebral hemorrhage (ICH) is a major public health problem accounting for 10–15% of all primary strokes^1^. Notably, deep-seated basal ganglia hemorrhage, the most common form of spontaneous ICH, is associated with a high mortality risk, resulting in death or dependency in >70% of the patients^1–4^. Variables such as advanced age, poor initial neurological state, large hematoma volume, and intraventricular extension are independent predictors of poor outcomes following spontaneous ICH^5–8^.

Recently, interest in the role of brain atrophy in reducing regional mass effect of space-occupying lesions has risen^9–12^. Brain atrophy may prevent an increase in intracranial pressure (ICP) and cerebral herniation in patients with large cerebral infarctions, by providing additional intracranial space for compensation^9–12^. However, the potential impact of brain atrophy on spontaneous ICH has not been clearly elucidated. While recent research suggests that reduced cerebral volume impedes functional recovery following supratentorial ICH^13^, no studies have reported the effect of brain atrophy specifically on the outcome of basal ganglia hemorrhage. Therefore, we investigated the contribution of brain atrophy to the favorable functional outcome of patients with moderate-volume basal ganglia hemorrhage.

## Methods

### Patient selection and data acquisition

We retrospectively reviewed 1003 consecutive patients with spontaneous ICH treated in our institute, between March 2006 and September 2016. Of these, 124 were included in this study, and all were diagnosed with moderate-volume (20–50 mL) basal ganglia hemorrhage, based on computed tomography (CT) scans. The remaining 879 patients were excluded for the following reasons: (1) hemorrhage associated with antecedent disease, including arteriovenous malformation, cavernous malformation, moyamoya disease, tumor bleeding, or hemorrhagic transformation of cerebral infarction (n = 73); (2) ICH at locations other than the basal ganglia (n = 331); (3) hemorrhage extension to the ventricular space (n = 124); (4) hematoma volume <20 mL (n = 246) or >50 mL (n = 73); (5) age >80 years (n = 18) or a premorbid bedridden state (n = 1); and (6) lost to follow-up (n = 6). Patients who underwent repeated surgery (n = 2), developed severe medical complications (n = 2), and whose relatives refused treatment (n = 3) were also excluded.

All the patients’ medical records from hospital charts and radiographic studies were reviewed. The functional outcome was evaluated using the Glasgow Outcome Scale (GOS) at 90 days after symptom onset. A GOS score of 4–5 was considered a favorable outcome, while 1–3 was considered unfavorable. Outcome measurement was performed during outpatient visits or via telephone interviews.

This study was approved by the institutional review board of Hanyang University Medical Center. Owing to the retrospective nature, the need for informed consent was waived.

### Image analysis

For most patients, hematoma volume was measured from initial CT scans; follow-up CT was used when hematoma expansion was detected. To estimate hematoma volume, we used the ABC/2 formula, where A represents the largest diameter on axial CT slices, B represents the diameter perpendicular to A on the same slice, and C represents the number of slices with a visible hematoma, multiplied by the slice thickness^14^. A volume of 20–50 mL was considered moderate^15^.

**Figure 1:**
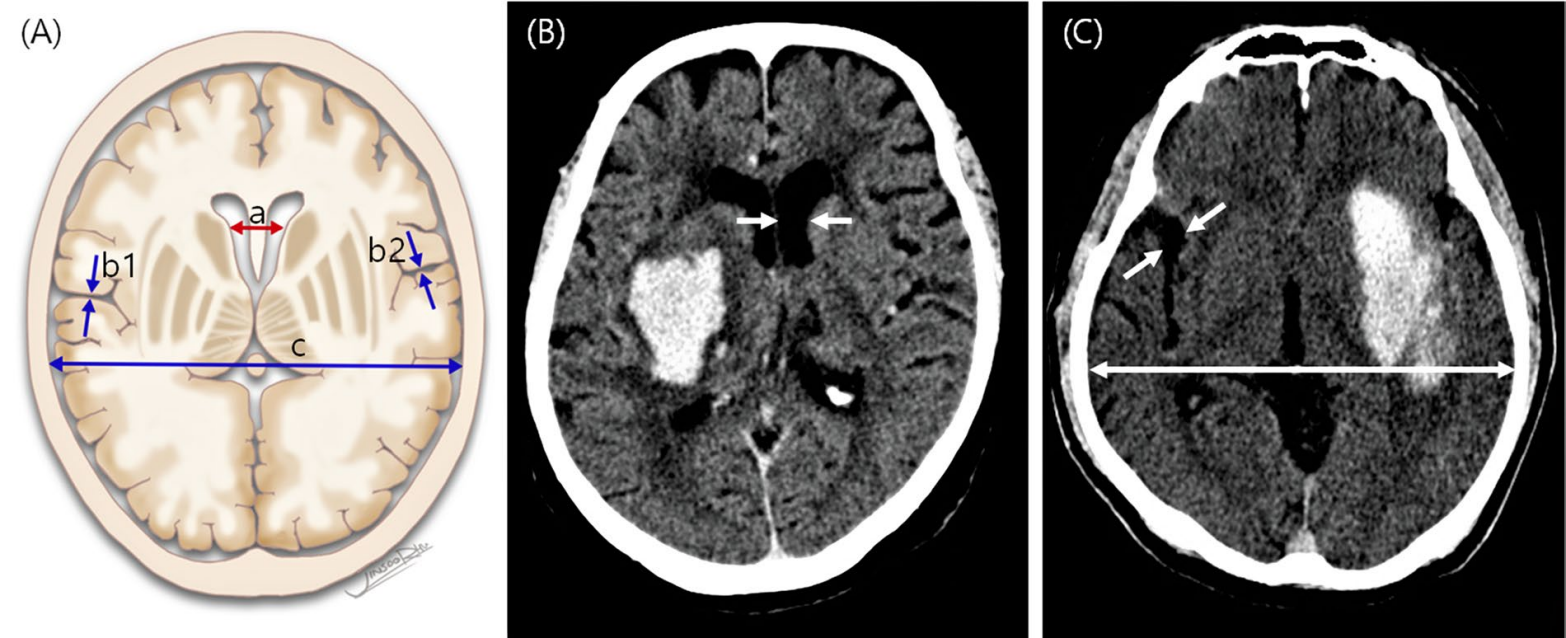
The intercaudate distance (ICD) and sylvian fissure ratio (SFR) as linear brain atrophy parameters. (**A**) Schematic figure showing the classic measurement technique for the ICD (a) and SFR [(b1 + b2)/2c]. The figure is adapted by permission from Jinsoo Rhu, the copyright holder. (**B** and **C**) Linear measurements from computed tomography images. The ICD is estimated by initial measurement of the hemi-ICD on the unaffected side and multiplication of the derived value by 2. For measurement of the SFR, the maximal width of the sylvian fissure on the unaffected side is divided by the transpineal inner table diameter.

We used the intercaudate distance (ICD) and sylvian fissure ratio (SFR) on CT scans as parameters of brain atrophy. The ICD, which represents central atrophy, was defined as the minimum distance between the caudate indentations on the frontal horns of the lateral ventricles^16,17^. The SFR, which represents cortical atrophy, was defined as the average maximum width of both sylvian fissures in the section where they appeared widest, divided by the transpineal inner table diameter (Fig. 1A)^16,17^. However, as hematoma on the morbid side can obscure measurements, only the unaffected hemisphere was assessed^17^. Therefore, the ICD was replaced by the hemi-ICD: the minimum distance between the caudate indentation on the unaffected side and the septum pellucidum, multiplied by 2 (Fig. 1B). The width of the sylvian fissure on the unaffected side only was used for calculating the SFR (Fig. 1C). These measurements were independently conducted by a neurosurgeon and a neuroradiologist, blinded to the clinical outcome. The mean value of each measurement was used to enhance precision.

### Patient treatment

All patients were hospitalized within 3 days of symptom onset, and received acute stage treatment in a neurosurgical intensive care unit. A CT scan was obtained for all patients upon admission. A second scan was obtained after 4–8 hours, unless the neurological status deteriorated. Systolic blood pressure was maintained <140 mmHg using antihypertensive agents. Osmotic diuretics (mannitol and glycerol) were administrated to control increased ICP. Prophylactic antiepileptic drugs were not used, and any anticoagulation or antiplatelet medications were discontinued for at least 5 days from the time of admission. Based on the Glasgow Coma Scale (GCS) score, hematoma volume, mass effect, and presence of herniation, surgical treatment involving stereotactic catheter insertion or hematoma evacuation with craniotomy was considered. All patients underwent a comprehensive rehabilitation program during or after hospitalization.

### Statistical analysis

All statistical analyses were performed using SPSS version 18.0 (SPSS, Chicago, Illinois). Student’s *t*-test or Mann–Whitney U test was used to compare continuous variables, and chi-square or Fisher’s exact test was used to compare categorical variables. Continuous variables were expressed as the mean ± standard deviation or median (interquartile ranges), while discrete variables were expressed as a count with percentage. Pearson’s correlation analysis was used to investigate the correlation between two continuous variables. To identify independent predictors of the functional outcome, backward logistic regression analysis was performed. Variables were considered for multivariate analysis only if they exhibited a P-value <0.2 in univariate analysis. A P-value <0.05 was considered statistically significant.

### Data availability

All data generated or analysed during this study are included in this published article (and its Supplementary Information files).

## Results

### Overall clinical outcome and group comparisons

Of the 124 patients (36 women; 55.7 ± 11.8 years) with moderate-volume basal ganglia hemorrhage, 74 (59.7%) showed favorable functional outcomes based on the GOS score at 90 days. Table 1 shows the baseline characteristics of the patients according to functional outcome. Brain atrophy parameters were significantly greater for the favorable group than for the unfavorable group. However, due to severe brain edema affecting the contralateral hemisphere, ICD and SFR measurements were not obtained for 7 and 3 patients, respectively.

**Table 1:** Baseline characteristics of patients with or without favorable functional outcome.

Variable	Favorable group (n = 74)	Unfavorable group (n = 50)	*P*-value
Age, years	54.5 ± 12.1	57.4 ± 11.3	0.187
Female sex	18 (24.3%)	18 (36.0%)	0.229
Medical history			
Hypertension	41 (55.4%)	24 (48.0%)	0.531
Diabetes mellitus	7 (9.5%)	3 (6.0%)	0.639
Other comorbidities	8 (10.8%)	7 (14.0%)	0.800
Previous ICH	5 (6.8%)	3 (6.0%)	1.000
Previous ischemic stroke	4 (5.4%)	4 (8.0%)	0.838
Smoking	30 (40.5%)	16 (32.0%)	0.438
Alcohol	48 (64.9%)	29 (58.0%)	0.559
BMI, kg/m^2^	23.52 ± 4.66	23.35 ± 2.89	0.828
Medication history			
Antihypertensive use	24 (32.4%)	13 (26.0%)	0.372
Antithrombotic use	5 (6.8%)	5 (10.0%)	0.753
Clinical features			
Time to treatment, min	75 (45–165)	60 (30–120)	0.228
Systolic BP, mmHg	170 (140–200)	169.5 (146–190)	0.838
Diastolic BP, mmHg	98 (80–110)	100 (86–110)	0.467
GCS score	13 (12–14)	10 (8–13)	<0.001
GCS score ≤ 8	2 (2.7%)	14 (28.0%)	<0.001
CT findings			
Dominant hemisphere	28 (37.8%)	30 (60.0%)	0.025
Hematoma volume, mL	29.4 (22.6–40.1)	36.2 (28.6–44.1)	0.004
ICD, mm	15.5 (11.7–18.0)	11.8 (9.6–13.8)	0.001
SFR	0.051 (0.039–0.064)	0.041 (0.032–0.055)	0.012
Midline shift, mm	3.9 ± 2.5	6.7 ± 3.3	<0.001
Treatment			0.001
Conservative treatment	30 (40.5%)	6 (12.0%)	
Catheter insertion	43 (58.1%)	36 (72.0%)	
Craniotomy	1 (1.4%)	8 (16.0%)	

BMI, body mass index; BP, blood pressure; CT, computed tomography; GCS, Glasgow Coma Scale; ICD, intercaudate distance; ICH, intracerebral hemorrhage; SFR, sylvian fissure ratio.

### Prognostic factors for favorable outcome

The results of the logistic regression analysis are summarized in Table 2. In multivariate analysis, GCS score at admission (odds ratio [OR], 1.311; 95% confidence interval [CI], 1.019–1.686), hematoma volume (OR, 0.941; 95% CI, 0.888–0.997), the ICD (OR, 1.207; 95% CI, 1.004–1.451), and the SFR (OR, 1.046; 95% CI, 1.007–1.086, per 0.001) values exhibited significant and independent correlations with a favorable functional outcome. Although the effect of age was not significant in univariate analysis, it was significantly associated with a favorable outcome in multivariate analysis (OR, 0.869; 95% CI, 0.812–0.931).

**Table 2:** Logistic regression analyses for predictors of a favorable functional outcome.

	Favorable outcome				
	Univariate analysis		Multivariate analysis		
	OR	*P*-value	OR	95% CI	*P*-value
Age	0.979	0.186	0.869	0.812–0.931	<0.001
History of hypertension	1.346	0.418			
Previous ICH	1.135	0.866			
Previous ischemic stroke	0.657	0.566			
Antihypertensive use	1.329	0.485			
Antithrombotic use	0.652	0.518			
Dominant hemisphere	0.406	0.016	0.904	0.301–2.718	0.857
GCS score	1.597	<0.001	1.311	1.019–1.686	0.035
Hematoma volume	0.947	0.006	0.941	0.888–0.997	0.041
ICD	1.204	0.001	1.207	1.004–1.451	0.045
SFR, per 0.001	1.034	0.002	1.046	1.007–1.086	0.021
Surgical treatment	0.200	0.001	0.374	0.080–1.742	0.210

CI, confidence interval; GCS, Glasgow Coma scale; ICD, intercaudate distance; ICH, intracerebral hemorrhage; OR, odds ratio; SFR, sylvian fissure ratio.

### Brain atrophy and clinical features

**Figure 2:**
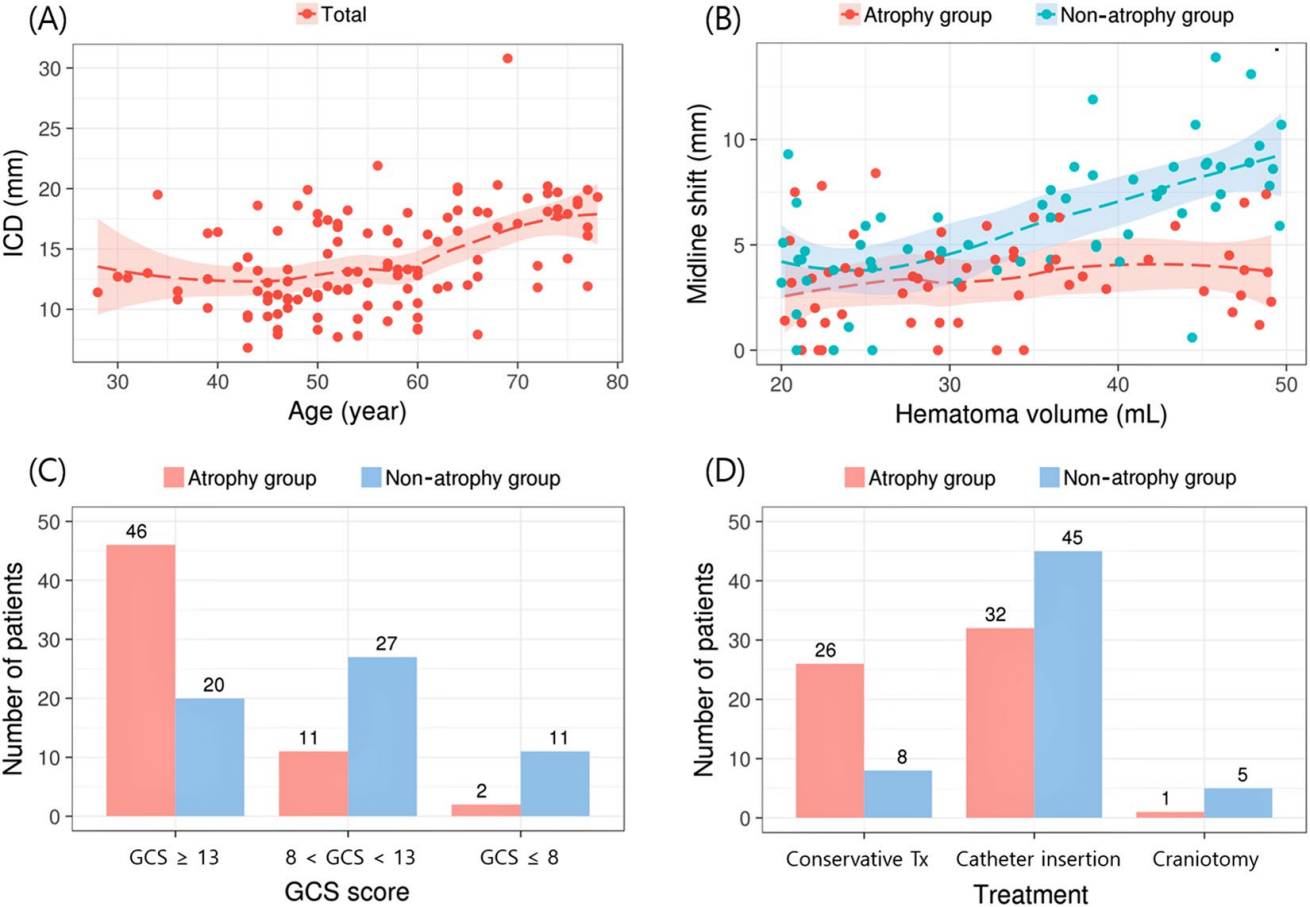
Clinical and radiological features of the atrophy and non-atrophy groups. (**A**) Scatterplot showing an increase in the intercaudate distance (ICD) with advancing age. The dotted line represents the Loess (locally weighted scatterplot smoothing) curve, and the shaded bands around the curve indicate 95% confidence limits. (**B**) Relationship between the hematoma volume and midline shift. (**C**) Distribution of the Glasgow Coma Scale (GCS) scores at admission for the two groups. (**D**) Treatment modalities for the two groups.

The ICD was applied to estimate the influence of brain atrophy on clinical features. With the median ICD value being used as a fiducial point, all patients (except 7 with missing ICD data) were dichotomized into two groups (the atrophy group, with an ICD ≥13.22 mm; and the non-atrophy group, with an ICD <13.22 mm). Clinical and radiographic features were compared between the two groups (Fig. 2 and Supplementary Table S1).

The mean age was significantly different between the atrophy and non-atrophy groups (60.5 ± 12.1 vs. 50.7 ± 10.1 years; P < 0.001), and the ICD increased with advancing age (Fig. 2A, correlation coefficient = 0.432; P < 0.001). Figure 2B presents the relationship between hematoma volume and midline shift. The mean midline shift was significantly smaller in the atrophy group than in the non-atrophy group (3.3 ± 2.2 vs. 6.1 ± 3.1 mm; P < 0.001). Additionally, there was no apparent correlation between the increase in midline shift and the hematoma volume in the atrophy group (correlation coefficient = 0.190; *P* = 0.150), whereas the midline shift was significantly augmented by the hematoma volume in the non-atrophy group (correlation coefficient = 0.631; P < 0.001). Figure 2C and D show the distribution of initial GCS scores and the treatment modalities used for the two groups. The admission GCS score was higher in the atrophy group than in the non-atrophy group (median, 14, interquartile range [IQR], 13–14 vs. 11.5, 9–13; P < 0.001). Moreover, the proportion of patients with a GCS score ≥13 was higher in the atrophy group than in the non-atrophy group (78.0 vs. 34.5%; P < 0.001), and patients in the former group exhibited a greater tendency of receiving nonsurgical treatment compared with those in the latter (44.1 vs. 13.8%; P < 0.001).

## Discussion

This study was performed to assess the contribution of advanced brain atrophy to favorable functional outcome in patients with moderate-volume basal ganglia hemorrhage. Several studies have found that brain atrophy prevents malignant outcomes by reducing the mass effect in patients with large cerebral infarctions^9–12^. Furthering these findings, the protective effect of brain atrophy against space-occupying lesions has been demonstrated in this study. Both the ICD and SFR were significantly correlated with a favorable outcome. This indicates that the relative increase in the intracranial volume reserve, caused by brain atrophy, may prevent an increase in ICP and subsequent brain herniation, thereby protecting against cerebral edema.

Conversely, Herweh *et al*. suggested cerebral atrophy as an independent risk factor for poor outcome following spontaneous ICH and reported that preexisting neurodegenerative changes may result in an unfavorable outcome in such cases^13^. This apparent discrepancy in results can be attributed to the difference in study design, in terms of patient inclusion. Herweh *et al*. included hemorrhages in any supratentorial region that exhibited a wider volumetric range and smaller mean volume (mean, 12.8; range, 0.3–153.4 mL) compared with those included in this study^13^. Since local mass effects have different impacts depending on their locations, swelling in the basal ganglia is more strongly associated with a poor outcome compared with swelling at other locations^9^. Consequently, superficially located smaller hematomas are considered to result in a less prominent mass effect compared with the lesions included in this study.

Previously identified prognostic factors (age, hematoma volume, and admission GCS score) are also well-reflected in our study^5–8,18,19^. Interestingly, age did not exhibit significant effect in univariate analysis, although it was strongly associated with clinical outcome^19^. This may be due to age-related differences in brain atrophy. As brain atrophy is mostly influenced by age^20,21^, it seems to counteract the worsening effect of advanced age on clinical outcome. Another study investigated the prognostic factors for spontaneous ICH using inclusion criteria similar to those used in this study, and likewise showed a weak correlation between age and clinical outcome in univariate analysis (P = 0.282)^15^.

The admission GCS scores and midline shift showed significant differences between the atrophy and non-atrophy groups. The initial GCS score is a powerful and independent prognostic factor for clinical outcome^22^. The GCS scores and proportion of patients with a GCS score ≥13 were significantly higher in the atrophy group than in the non-atrophy group. Midline shift resulting from brain edema is a major determinant of cerebral herniation and a malignant outcome^23,24^. In this study, the midline shift was smaller in the atrophy group than in the non-atrophy group. Moreover, the increase in midline shift, in proportion to the hemorrhage volume, was smaller in the atrophy group. Therefore, preexisting brain atrophy may provide protection against an unfavorable outcome by decreasing ICP and preventing midline shift in the acute phase, despite possible neurodegeneration unrelated to injury. In the atrophy group, however, involvement of the dominant hemisphere was less frequent (33.9% vs. 55.2%; *P* = 0.003) and hematoma volumes were relatively small (median, 29.5, IQR, 23.4–36.8 vs. 35.9, 24.9–44.4 mL; *P* = 0.141) compared with those in the non-atrophy group. This may have influenced the disparity in other variables between the two groups.

In the present study, only patients with a hematoma volume of 20–50 mL were included. While the treatment and prognosis of moderate-volume hemorrhage have the greatest uncertainty^4^, a hematoma volume <20 mL manifests in little mass effect, and bleeding >50 mL correlates with high mortality, even with adequate treatment^18^. Hence, the compensatory effect of brain atrophy would not be obvious in either of these groups. Additionally, patients with hemorrhage extending to the ventricles were excluded because this may influence the outflow of cerebrospinal fluid (CSF) and measurement of the ICD.

We used the ICD and SFR as linear brain atrophy parameters. However, in some patients, hematoma obscured measurements in the contralateral hemisphere. Although 7 patients were excluded from atrophy measurement because the contralateral ventricle and/or sylvian fissure were already collapsed, mild deformation was seen in a small number. This subtle change may have caused inaccuracy in measurements. Several studies have used a semiautomatic method to perform volumetric analysis of the intracranial and CSF spaces^11,13^. While this process improves measurement accuracy, it requires a specialized assessment technique and is not easily applicable in clinical practice. Therefore, we used linear measurements that allow simple and reliable assessment of brain atrophy, with considerable interobserver agreement^16,17^.

This study has the following limitations: firstly, since the study was retrospective, selection bias cannot be excluded, and some data were unobtainable; secondly, the number of enrolled patients was relatively small, which can lower the statistical power, because inclusion was limited to patients with a precise hemorrhage size and location; thirdly, treatment modalities were varied; this heterogeneity may have affected the results; finally, as mentioned earlier, the measurement of brain atrophy may not be precise in some patients due to severe brain edema affecting the contralateral hemisphere.

In conclusion, preexisting brain atrophy exhibits a protective effect in patients with basal ganglia hemorrhage. This suggests that brain atrophy, along with age, hematoma volume, and initial neurological state, may provide prognostic information regarding the functional outcome following moderate-volume basal ganglia hemorrhage.

## Electronic supplementary material

Supplementary Table S1
