# Supplementary material for: Impact of brain atrophy on 90-day functional outcome after moderate-volume basal ganglia hemorrhage
Source: Sci Rep. 2018 Mar 19;8:4819. doi: 10.1038/s41598-018-22916-3 (PMC5859038; doi:10.1038/s41598-018-22916-3)
Supplement: Supplementary file 1 — Supplementary Table S1 [file 41598_2018_22916_MOESM1_ESM.pdf]

Supplementary information

Impact of brain atrophy on 90-day functional outcome after moderate-volume basal ganglia hemorrhage

Sae Min Kwon[1], Kyu-Sun Choi[1], Hyeong-Joong[1], Yong Ko[1], Young-Soo Kim[1], Koang-Hum Bak[1], Hyoung-Joon Chun[1], Young-Jun Lee[2], Ji Young Lee[2]

Departments of [1]Neurosurgery and [2]Radiology, College of Medicine, Hanyang University, Seoul, Korea

Supplementary Table S1. Comparison of clinical and radiographic features between patients with brain atrophy and those without brain atrophy

Variable	Atrophy group (ICD ≥13.22, n=59)	Non-atrophy group (ICD <13.22, n=58)	*P*-value
Age, year	60.5±12.1	50.7±10.1	<0.001
Female sex	16 (27.1%)	19 (32.8%)	0.642
GCS score	14 (13–14)	11.5 (9–13)	<0.001
GCS score ≥13	46 (78.0%)	20 (34.5%)	<0.001
8< GCS score <13	11 (18.6%)	27 (46.5%)	
GCS score ≤8	2 (3.4%)	11 (19.0%)	
CT findings			
Dominant hemisphere	20 (33.9%)	32 (55.2%)	0.033
Hematoma volume, mL	29.5 (23.4–36.8)	35.9 (24.9–44.4)	0.141
Midline shift, mm	3.3±2.2	6.1±3.1	<0.001
Treatment			<0.001
Conservative treatment	26 (44.1%)	8 (13.8%)	
Catheter insertion	32 (54.2%)	45 (77.6%)	
Craniotomy	1 (1.7%)	5 (8.6%)	

Values are expressed as the mean±standard deviation, median (interquartile range), or number (percentage).

CT, computed tomography; GCS, Glasgow Coma Scale; ICD, intercaudate distance.
